# PD17 - Food allergy profile in late adolescence in a tertiary healthcare facility

**DOI:** 10.1186/2045-7022-4-S1-P17

**Published:** 2014-02-28

**Authors:** Anastasia Georgountzou, Anastassia Karamouza, Stavroula Giavi, Eirini Roumpedaki, Nikolaos Douladiris, Emmanouil Manousakis, Nikolaos G Papadopoulos

**Affiliations:** 1 Allergy Department, 2nd Pediatric Clinic, University of Athens, Athens, Greece

## Background and aim

Food allergies are increasing and food allergic adolescents are at higher risk of anaphylaxis. We report on food allergy (FA) profile in late adolescence in a tertiary healthcare facility.

## Methods

A medical record review was performed including patients born before 1999, who had visited our allergy department during the last seven years and had been followed-up in the last 6 months. Diagnosis of FA was based on reported immediate reactions to foods and positive SPT/ food-specific serum IgE and/or positive food challenge.

## Results

Among 56 patients (46 male, age range 14-22,5 years, median 16,6 years) 55% had positive family history of atopy, 64% reported atopic dermatitis and 80% asthma and/or allergic rhinitis. 1/3 developed their first reaction to a food allergen during infancy, the main allergens being egg (11%), milk (9%) and fish (7%).32% first experienced FA as preschoolers, 27% between 6-12 years and only 11% in adolescence. In 21/56 patients the first reaction was anaphylaxis. All subjects became tolerant to milk and egg before adolescence. The most common allergens in late adolescence were nuts (including peanut) (55%), fruits (25%), fish (23%) and sesame seed (9%). Nut allergy appeared in 48,4% (15/31 patients) in preschool years, in 25,8% between 6-12 years and in 25,8% during adolescence. The rates for fish allergy apparition were 69,2%(9/13 patients), 23,1% and 7,7%, respectively. Fruit allergy emerged in most patients between 6 - 12 years old. Only 2 patients became tolerant to fish and 3 to nuts before/during adolescence. Over half of the patients experienced at least 1 anaphylactic reaction and 20% had 2 or more. An adrenaline auto-injector was prescribed to 80% of the patients. Nevertheless, only 18% reported carrying it at all circumstances and 20% occasionally.

## Conclusion

Nut, fish and fruits are the most common food allergens in late adolescence, the first two arising predominantly before school age and fruit allergy during school age years. Less than half of our patients report satisfactory compliance to medical advice regarding access to adrenaline auto-injectors.

